# Atrial Septal Defect and Heart Rhythm Disorders: Physiopathological Linkage and Clinical Perspectives

**DOI:** 10.3390/biomedicines13102427

**Published:** 2025-10-04

**Authors:** Adriana Correra, Alfredo Mauriello, Matilde Di Peppo, Antonello D’Andrea, Vincenzo Russo, Giovanni Esposito, Natale Daniele Brunetti

**Affiliations:** 1 Cardiology Unit, Department of Cardiology, University of Foggia, 71122 Foggia, Italy; matilde.dipeppo@gmail.com (M.D.P.); natale.brunetti@unifg.it (N.D.B.); 2 Division of Cardiology, Institute National Cancer—IRCCS—Foundation “G. Pascale”, 80131 Napoli, Italy; alfredo.mauriello93@libero.it; 3 Cardiology and Intensive Care Unit, Department of Cardiology, “Umberto I” Hospital, 84014 Nocera, Italy; antonellodandrea@libero.it; 4 Cardiology Unit, Department of Medical and Translational Sciences, University of Campania “Luigi Vanvitelli”, Monaldi Hospital, 80131 Naples, Italy; vincenzo.russo@unicampania.it; 5 Cardiology Unit, Department of Advanced Biomedical Sciences, University of Naples Federico II, 80131 Naples, Italy; giovanni.esposito2@unina.it

**Keywords:** atrial septal defect, congenital heart disease, electrocardiogram, heart rhythm disorders

## Abstract

An atrial septal defect (ASD) is the most common congenital heart defect (CHD) diagnosed in adulthood. It is characterized by significant anatomical heterogeneity and complications that evolve over time. While often asymptomatic in children, the signs of adverse effects of ASD increase with age, including a greater risk of heart failure, stroke, atrial fibrillation (AF), and reduced life expectancy. ASD is traditionally considered a right-heart lesion due to long-term complications such as arrhythmias, right-sided heart failure, thromboembolism, and, in a subset of patients, pulmonary arterial hypertension (PAH). The pathophysiology of atrial shunts also affects the left heart due to volume overload and adverse ventriculo-ventricular interaction. Early diagnosis of interatrial septal anomalies is essential to prevent hemodynamic consequences and/or thromboembolic events. Electrocardiographic (ECG) findings play a crucial role in this early diagnosis. This narrative review aims to update clinicians on the latest evidence regarding the pathophysiological link between ASD and cardiac rhythm disorders, the nuances of optimal diagnostics, treatment options (surgical, interventional, pharmacological), and the need for long-term follow-up for patients with ASD. The review will determine the risk of conduction disorders compared to a healthy population and to compare the prevalences of conduction disorders, mortality, and pacemaker use in patients with closed ASDs versus those with open ASDs.

## 1. Introduction

The atrial septal defect (ASD) is the third most common congenital heart anomaly (CHA), representing the most frequent cardiac malformation diagnosed in adulthood [1]. The defect can be located in different positions in the interatrial septum, or as interatrial communication, with ostium secundum defects being the most frequent (about 80% of all ASDs). Other types include ostium primum defects (partial AVSD, about 15%), sinus venosus defects (superior and inferior, 5–10%), and, more rarely, coronary sinus defects (<1%) [2]. Table 1 summarizes several types of defects.

Most children with isolated ASD are asymptomatic [2]. However, signs of adverse effects of an ASD increase with age [3]. Patients with ASD have an increased risk of developing heart failure, stroke, atrial fibrillation (AF), and pneumonia, as well as a reduced life expectancy, regardless of closure in childhood or adulthood [3,4]. Consequently, patients with repaired ASD, long considered completely healthy after defect correction, deserve renewed attention [5].

Recent studies have shown that patients with familial ASD have an increased risk of sudden cardiac death. Some of these patients carry a mutation in the NKX2-5 gene, which is essential for cardiac development and atrioventricular node maturation [6,7]. Alterations in this gene can lead to conduction abnormalities, particularly atrioventricular block, with potentially fatal outcomes [8].

Furthermore, a significant shunt can lead, over the years, to right ventricular dilation, reduced reserve function, myocardial cell hypertrophy and fibrosis, as well as cellular damage [9]. This dilation lengthens myocardial fibers, potentially altering the conduction of electrical impulses within the myocardium and leading to atrial tachyarrhythmias or bradyarrhythmias [10].

Given the potential alteration of electrical impulse conductance in the myocardium and the association between ASD and NKX2-5, TBX5, and PRRX1 gene mutations, this review aims to investigate the clinical prevalence of atrioventricular block, bundle branch block, fascicular block, bradycardia, and the need for pacemakers in patients with ASD. The objective is to determine the risk of conduction disorders compared to a healthy population and to compare the prevalences of conduction disorders, mortality, and pacemaker use in patients with closed ASDs versus those with open ASDs. 

AT: atrial tachycardia; IRBBB: incomplete right bundle branch block; LAFB: left anterior fascicular block.

## 2. Pathophysiology of Atrial Septal Defect and Electrophysiological Impact

### 2.1. Left-to-Right Shunt and Cardiac Remodeling

ASD typically results in a left-to-right shunt, whose direction and magnitude are determined by the size of the defect, relative atrial pressures related to the compliance of both ventricles, and changes over time [11]. Small defects (generally <10 mm) are associated with a reduced shunt and minimal to no enlargement of right heart structures. However, larger and long-standing shunts lead to right atrial and ventricular dilation, stretching of myocardial cells, and damage/impairment over time [12]. Increased pulmonary blood flow can trigger a pathological mechanism due to shear stress, causing activation of pulmonary endothelial cells and activation of growth factors, vasoconstrictors, and smooth muscle hypertrophy, thereby contributing to the development of pulmonary arterial hypertension (PAH) [13]. At the same time, the pathophysiology of shunts at the atrial level also influences the left heart due to volume overload and adverse ventriculo-ventricular interaction [14].

Cardiac remodeling after defect closure is evident almost immediately, although the remodeling process appears to continue for at least one year. The extent of cardiac remodeling, i.e., the decrease in right heart volumes with an increase in left ventricular filling, is inversely correlated with age at the time of closure. Persistent right heart dilation and residual functional tricuspid regurgitation are more prevalent in elderly patients with late ASD closure and are associated with elevated brain natriuretic peptide levels and right ventricular dysfunction [12].

### 2.2. Electrophysiological Basis of Arrhythmias

The dilation of myocardial fibers can alter the conduction of electrical impulses within the myocardium, leading to atrial tachyarrhythmias or bradyarrhythmias [15]. The electrophysiological properties of atrial myocytes are altered, with an increase in intra-atrial conduction time, likely due to a combination of interstitial fibrosis and chamber enlargement [16]. The conduction properties of the sinus node may also be altered in the pre-operative state. Impairments in the NKX2-5 gene lead to conduction abnormalities, particularly atrioventricular block, which can ultimately have a fatal outcome [15].

### 2.3. QT and RR Interval Variability

ASD causes changes in cardiac hemodynamics that depend on the size of the defect and the volume of the shunt [8]. This pathological change influences sinus node automaticity and myocardial depolarization and repolarization, with the potential to evoke arrhythmogenic substrates. QT interval variability (QTVI) and Variability Ratio (VR) are indices that reflect myocardial repolarization instability and heterogeneity [17]. A retrospective study in 38 children with ASD (mean age, 2.2 ± 1.9 years; mean left-to-right shunt ratio, 2.1 ± 0.70) revealed a strong positive correlation between the Qp/Qs ratio (pulmonary/systemic blood flow) and both VR (r = 0.662, *p* < 0.0001) and QTVI (r = 0.808, *p* < 0.0001) [17]. Analysis of cardiac cycle and repolarization parameters in pediatric patients with ASD showed multiple differences compared to controls. The standard deviation of all regular RR intervals (SDNN) and heart rate variability (HRv) were significantly lower in patients with high-shunt ASD (Qp/Qs ≥ 2.0) compared to patients with low-shunt ASD (Qp/Qs < 2.0) or the control group. Conversely, the standard deviation of QT intervals (SDQT) and QT variance (QTv) were significantly higher in the high-shunt ASD group. These repolarization indices provide information on the alteration of autonomic sinus node control and the pathophysiology of myocardial repolarization and can be used as indicators of the shunt [17].

Right ventricular remodeling, characterized by paradoxical interventricular septal movements, compromises left ventricular filling, leading to a reduction in left ventricular ejection volume and cardiac output in children with ASD, and an increase in left-to-right shunt with increased sympathetic nervous activity [18]. The high lability of repolarization is directly associated with sympathetic cardiac activation [19].

## 3. Rhythm and Conduction Abnormalities Associated with ASD

Arrhythmias and conduction disturbances are well-described in patients with ASD [20].

### 3.1. Atrial Tachyarrhythmias

Atrial tachyarrhythmias are commonly observed in patients with ASD, regardless of the ASD type. AF and atrial flutter (AFL) are relatively rare in childhood but become more prevalent with increasing age at the time of repair or defect closure. The prevalence of atrial arrhythmias is also correlated with shunt size, hemodynamic complications such as pulmonary hypertension, and other comorbidities [21].

Dilation of atrial and ventricular tissues can affect conduction properties. Surgical incisions and inserted devices can add to the disturbances, causing a higher prevalence of conduction abnormalities [22].

A Danish nationwide study [23], included 151 patients (mean age 32 years), examined the hidden burden of atrial and ventricular arrhythmias in asymptomatic adult patients with small, unrepaired ASDs, without a previous diagnosis of AF. The results showed that one in five patients had arrhythmia, although most patients had experienced spontaneous closure of their ASD. The most common arrhythmias were supraventricular tachycardias (SVT) (16%), particularly focal atrial tachycardia (FAT) (21 patients), followed by non-sustained ventricular tachycardia (NSVT) (8%). The presence of arrhythmia was associated with an increased right ventricular/left ventricular (RV/LV) diastolic area ratio and older age. This suggests that myocardial structural changes induced by early life volume overload may be irreversible and provide the substrate for subsequent arrhythmias, even in the absence of hemodynamic residuals. The prevalence of paroxysmal AF was low (1.3%) in this asymptomatic group. Still, it is conceivable that the high incidence of non-sustained atrial arrhythmias found may be a precursor to the development of AF at a later age, as FATs can trigger other atrial arrhythmias.

#### 3.1.1. Post-ASD Closure

The incidence of atrial tachyarrhythmias decreases after ASD closure [24]. Still, the recurrence rate can remain significant, particularly in patients who underwent closure at an older age, with larger shunts, or with other comorbidities. After surgical closure, additional intra-atrial macro-reentry circuits may exist, most commonly around the atriotomy site and the ASD patch edges [15].

A meta-analysis of 25 studies [25] concluded that percutaneous ASD closure was not associated with a reduction in all atrial arrhythmia or AF prevalence post-closure (OR 0.855, 95% CI 0.672 to 1.087, *p* = 0.201 and OR 0.818, 95% CI 0.645 to 1.038, *p* = 0.099, respectively). Some studies [26] suggest that closure at a younger age (e.g., <40 years) offers some protection against AF. Age at closure is an essential predictor of postoperative arrhythmias. Intra-atrial reentrant tachycardia (IART) is often the result of surgical palliation of CHDs, due to scar tissue that can serve as a possible focus. However, device closure may offer protection against the development of arrhythmias, particularly in patients under 55 years of age.

Atrial arrhythmias are significantly increased in patients with secundum ASD, with an incidence of about 10% in ASD patients under 40 years old and about 20% in ASD patients aged 40 years or older. The data are not specific to the shunt type [25].

Overall, a meta-analysis of 25 studies did not demonstrate a reduction in the prevalence of atrial arrhythmias after percutaneous closure in adults (OR 0.855, *p* = 0.201), and no significant decrease in prevalence was found for AF (OR 0.818, *p* = 0.099) [25].

Age modifies the effects of closure on the risk of atrial arrhythmias. A weak reduction in the prevalence of both all atrial arrhythmias (OR 0.77, *p* = 0.032) and AF alone (OR 0.760, *p* = 0.024) after ASD closure was observed only in patients between 40 and 60 years old. The beneficial effect became non-significant in the subgroup of patients older than 60 years (atrial arrhythmias: OR 0.822, *p* = 0.242; AF: OR 0.83, *p* = 0.266) [25].

Regarding the effects on pulmonary hypertension, the weak benefit was also observed in the subgroup aged between 40 and 60 years [25].

#### 3.1.2. Atrioventricular Septal Defect (AVSD) and Atrial Arrhythmias

The risk of atrial arrhythmias in adult patients with AVSD is considerable, with over half of patients developing at least one atrial arrhythmia by age 60. In a multicentric cohort of 391 adult patients with a mean age of 36.3 ± 16.3 years and a mean follow-up of 17.3 ± 14.2 years with AVSD, IART/FAT is the most common arrhythmia until age 45, after which AF overtakes it. Independent risk factors for atrial arrhythmias include age (OR: 1.4 per 5-year increment), number of cardiac surgeries (OR: 4.1), left (OR: 3.1) and right (OR: 4.1) atrial dilation, and moderate to severe left atrioventricular valve insufficiency (OR: 3.7). The type of AVSD and age at repair are not associated with the risk of atrial arrhythmias. The occurrence of atrial arrhythmias is associated with pacemaker implantation, heart failure, and cerebrovascular events [27].

### 3.2. Bradyarrhythmias and Conduction Disturbances

Both sinus node abnormalities and atrioventricular (AV) node conduction abnormalities have been reported in patients with ASD [12].

#### 3.2.1. Atrioventricular Block (AVB)

First-degree AV block is standard in many patients with ASD, secondary primarily to prolonged intra-atrial conduction time. Higher-grade AV node conduction abnormalities are more frequently observed in patients with ostium primum type ASD [28]. This is likely due to the displacement of the AV node and His bundle and the proximity of the defect to these structures. AVB in these patients can develop spontaneously over time without surgical or procedural intervention. Post-surgical AVB can also develop due to injury to the conduction system during patch repair, although this is rare with modern surgical techniques [29]. Patients with surgically or catheter-closed ASDs showed a higher prevalence of atrioventricular block (6.4% vs. 0% in unclosed ASDs), incomplete right bundle branch block (IRBBB), and left anterior fascicular block (LAFB) compared to patients with unclosed ASDs. The prevalence of AVB was more than 60 times higher and complete right bundle branch block (CRBBB) was 4 times higher in patients with ASD over 25 years old compared to control cohorts. The higher prevalence of AVB among patients with catheter closure (13%) compared to surgical closure (5%) could be due to impedance of the conduction system by the device or older age at the time of closure [15].

#### 3.2.2. Right Bundle Branch Block (RBBB)

The most frequent finding is incomplete right bundle branch block (IRBBB) (40.1% of patients). This is due to delayed depolarization of the thickened right ventricular outflow tract and possibly right ventricular volume overload. The presence of an RSR’ pattern in V1-V3 with QRS duration <120 ms defines IRBBB. IRBBB is significantly more frequent in patients with ASD (56% vs. 5% in controls). The RSR’ pattern in V1 has a specificity of 80% and a sensitivity of 36.1% but alone is insufficient to diagnose an ASD [30]. One prospective study included 87 patients [31] showed that interatrial septal anomalies were present in 80.5% of patients with RBBB, with patent foramen ovale (PFO) as the most prevalent disorder (39.02%), followed by atrial septal aneurysm (ASA) (21.9%) and ASD (19.5%). The right heart chambers (right atrium and ventricle) were significantly larger in the RBBB group. Interestingly, IRBBB could regress or evolve into CRBBB over time. Although the presence of IRBBB in children is considered a normal finding and not an anomaly, CRBBB was more frequent in adults, while IRBBB was primarily present in preschool age [15].

#### 3.2.3. Left Anterior Fascicular Block (LAFB)

The prevalence of LAFB is 3.7% in patients with ASD and is significantly higher in closed defects (5.8% vs. 0.7% in unclosed ones) [15].

#### 3.2.4. Sinus Node Dysfunction

Sinus node dysfunction has been reported in patients with ASD [32]. The risk of developing sinus node dysfunction is correlated with larger shunt sizes and older age at closure. In patients with superior sinus venosus ASD, the proximity of the defect to the crista terminalis and the sinus node presents a higher risk. Older surgical techniques reported high rates (10–15%), while modern techniques like the Warden procedure or modified double-patch technique show a much lower incidence (1–2%) [32].

#### 3.2.5. Pacemaker

In the Danish Registry [15], ten (3.4%) of 297 patients with ASD required a pacemaker, with a mean age at implantation of 32 years. The main reasons were AVB (6 patients) and sinus node dysfunction (4 patients). Pacemaker implantation was performed, in most cases, many years after ASD treatment. Notably, seven ASD patients required a pacemaker before age 47, considering that the average age for the first pacemaker implantation in Denmark is 76 years. Patients with surgically or catheter-closed defects showed a greater need for pacemakers. Patients with AVSD and atrial arrhythmias also have a significantly higher incidence of pacemaker implantation (41.8% vs. 8.5%). Table 2 summarizes the features of patients with pacemaker implantation.

## 4. Diagnosis of Arrhythmias and Conduction Disturbances in ASD

The diagnosis of ASD and its arrhythmic complications relies on a multimodal approach, including non-invasive tools and advanced imaging techniques. Table 3 includes ECG clues to ASD by type.

### 4.1. 12-Lead Electrocardiogram

The standard 12-lead ECG remains a first-line diagnostic tool for identifying abnormalities associated with ASD.

#### 4.1.1. IRBBB and Right Axis Deviation

These are common findings. IRBBB is due to delayed depolarization of the thickened right ventricular outflow tract and likely right ventricular volume overload. In one study, IRBBB was present in 56% of ASD patients compared to 5% in the control group, with a specificity of 95% [15]. In conclusion, this is supportive sign rather than pathognomonic for diagnosis.

#### 4.1.2. Tall P Wave

Indicative of right atrial enlargement [15].

#### 4.1.3. Crochetage Sign (Notched R Wave)

A notch near the apex of the R wave in the inferior limb leads (II, III, aVF). It is an independent ECG sign of ostium secundum type ASD and correlates with the severity of the left-to-right shunt and ASD size [11]. Its presence in only one lead has a specificity of 92.6% and sensitivity of 73.1%; if present in all three inferior leads, specificity reaches 100%. This sign may disappear after surgical correction of the ASD. Its presence, especially with an RBBB, increases diagnostic specificity, but this is supportive rather than pathognomonic for diagnosis [30].

#### 4.1.4. Defective T Wave (DTW)

Defined as a horizontal or inverted shift in the proximal T wave segment in the right precordial leads. The coexistence of IRBBB and DTW has shown 100% specificity and 87.1% sensitivity for ASD diagnosis [35].

#### 4.1.5. Inverted P Waves

In the inferior leads, suggest ectopic atrial activity or sinus node dysfunction, as can be seen in sinus venosus defects [15].

#### 4.1.6. Left Axis Deviation and Atrioventricular Node Delay

Can be observed in ostium primum defects [15].

#### 4.1.7. Slightly Prolonged QRS with rSr’ or rsR’ Pattern

This is often observed and is believed to be the result of right ventricular volume overload rather than true transmission delay [15].

### 4.2. Holter Monitoring

7-day Holter monitoring can provide more accurate and precise data on asymptomatic and paroxysmal conduction disturbances, which might otherwise be missed, leading to an underestimation of the prevalence of these disturbances [36]. One study revealed a hidden burden of atrial and ventricular tachyarrhythmias in asymptomatic adult patients with small, unrepaired ASDs, even after spontaneous closure of the defect [23].

## 5. ASD Diagnosis

### 5.1. Echocardiography (TTE, TEE, 3DE, ICE)

Echocardiography remains the first-line imaging modality for the evaluation of patients with ASD [11].

### 5.2. Transthoracic Echocardiography (TTE)

Provides essential information on defect size and location, shunt direction, right heart dilation, and paradoxical interventricular septal motion during diastole, suggestive of hemodynamic significance. It also allows for estimation of pulmonary arterial pressure (PAP) [11].

### 5.3. Transesophageal Echocardiography (TEE)

Represents a complementary method, particularly useful in patients with suboptimal transthoracic windows and/or sinus venosus defects associated with anomalous pulmonary venous drainage. It offers essential intraprocedural imaging for catheter-based ASD closure and to assist with stent closure of sinus venosus defects [11].

### 5.4. Three-Dimensional Echocardiography (3DE)

This is a well-established complementary modality for ASD evaluation. It offers right or left “en face” views with clear visualization of the defect, especially if unusually shaped, fenestrated, and/or multiple defects, and delineation of surrounding tissue borders and relationship to adjacent structures. Real-time 3DE guidance during defect closure allows imaging of the catheter delivery process [11].

### 5.5. Intracardiac Echocardiography (ICE)

In some centers, it has largely replaced TEE for ASD closure. ICE can be performed by the primary operator of the interventional procedure under conscious sedation, eliminating the risk of esophageal trauma. A 3D-volumetric ICE system has recently been developed with potential for greater anatomical information [11].

### 5.6. Advanced Imaging (CMR, CT, 3D Printing, Holograms)

#### Cardiac Magnetic Resonance (CMR) and Computed Tomography (CT)

Allowing comprehensive imaging of all forms of atrial communications, especially when echocardiography leaves unresolved questions regarding the presence, location, size, or physiology of a pre-tricuspid communication [37]. CMR is the gold standard for ventricular volume and function measurements. Phase-velocity flow mapping allows accurate quantification of the Qp/Qs ratio. It is an important diagnostic tool in sinus venosus defects, particularly inferior ones, and for anomalous pulmonary venous drainage, which often hinder echocardiographic evaluation [11].

### 5.7. 3D Printing, Computational Modeling, and Holograms

These modalities are increasingly used to delineate the anatomy of sinus venosus defects, especially given their heterogeneity, and to enable therapeutic simulation and procedural planning [38]. Holographic models in augmented reality allow the clinician to navigate the model to examine procedural feasibility, post-device implantation anatomical relationships, and overall outcome [11].

### 5.8. Cardiac Catheterization

It is not routinely required for the diagnosis of ASD, but it is indispensable in patients with pulmonary hypertension (PH), left heart dysfunction, or risk factors for coronary artery disease. It allows for an invasive assessment of pulmonary vascular resistance (PVR) and responsiveness to vasodilators, guiding decisions on defect closure and pharmacological therapy for PH [11].

## 6. Clinical Management of Atrial Septal Defect and Associated Arrhythmias

The management of patients with ASD, especially in the presence of rhythm disturbances, requires a multidisciplinary and personalized approach, taking into account anatomical, hemodynamic, and patient age specificities.

### 6.1. Indications for ASD Closure

ASD closure is indicated in the presence of symptoms or right heart volume overload.

Closure aims to reduce right heart volume overload and, consequently, prevent or mitigate atrial remodeling and arrhythmias [23].

Early closure, ideally before 25 years of age, is associated with excellent long-term outcomes and normal survival. Morbidity increases with advanced age at closure. However, symptomatic benefits and improvements in quality of life are observed in all age groups, even in elderly patients. One study found that none of the patients operated before the age of 25 developed preoperative AF or AFL, while the percentage rose to 59% in those operated after 41 years of age. Postoperative arrhythmias are also less frequent in patients operated on at a younger age [39].

### 6.2. Methods of ASD Closure

#### 6.2.1. Transcatheter Closure

This is the treatment of choice for most ostium secundum ASDs, offering excellent results with low complication rates, shorter hospital stays, and faster recovery compared to surgery. The procedure is guided by fluoroscopy and TEE (or ICE). Various occluder devices exist, based on shape-memory alloys, which can be self-centering or adaptable to the defect’s waist (e.g., Amplatzer Septal Occluder, Figulla Flex II ASD Occluder, Cardioform ASD). Limitations include insufficient margins, multiple defects, or extremely large defects. Emerging techniques use covered stents for the closure of superior sinus venosus defects associated with anomalous pulmonary venous drainage [40].

#### 6.2.2. Surgical Closure

This is the standard treatment for sinus venosus, ostium primum, and coronary sinus defects, as well as for ostium secundum ASDs unsuitable for transcatheter closure. Historically, procedures were performed via median sternotomy, but currently, minimally invasive techniques (partial mini-sternotomy, thoracotomy) or robot-assisted techniques are preferred to reduce aesthetic and psychological impact, while maintaining similar outcomes in terms of morbidity and mortality [41].

### 6.3. Management of Arrhythmias

The management of arrhythmias in patients with ASD must consider the multifactorial nature of their onset and the presence of structural alterations [39].

#### 6.3.1. Transcatheter Ablation:

Pre-closure: Ablation of SVTs, particularly those with a left-sided substrate, should be considered before ASD closure to avoid complications related to transseptal puncture through a patch or device [42]. For patients with AF and a newly diagnosed ASD that meets closure criteria, catheter ablation should be performed before transcatheter closure [39].

Post-closure: Atrial tachyarrhythmias may persist or recur after closure. Macro-reentry circuits, often around atriotomy sites or patch edges, are frequently amenable to transcatheter ablation [43]. However, transseptal puncture can be complex in the presence of patches or devices [39].

#### 6.3.2. Antiarrhythmic Drugs and Cardioversion

Medical treatment of arrhythmias in ASD patients is similar to that in the general population, with appropriate consideration for antiarrhythmic drugs and electrical cardioversion [39].

#### 6.3.3. Anticoagulation

For patients with AF, thromboembolic prophylaxis is guided by risk scores (CHA2DS2-VASc and HAS-BLED). Direct oral anticoagulants may be considered, although data on their use in CHDs are (32 years) compared to the general population (76 years), suggesting a need for long-term follow-up for the onset of bradyarrhythmias [44]. Additionally, a meta-analysis involving 2796 patients with adult congenital heart disease (ACHD) suggests that in patients with ACHD, DOACs appear to be equally effective and safe compared to VKAs [45,46].

#### 6.3.4. Cardiac Remodeling After Defect Closure and Pulmonary Hypertension

Reverse remodeling of the right and left heart, including electrical changes, is evident almost immediately after closure and continues for at least one year. The extent of this remodeling is inversely correlated with age at closure. Persistent right heart dilation and residual functional tricuspid regurgitation are more prevalent in elderly patients with late closure [41]. PH is a complication of ASD, whose risk increases with age [41].

## 7. Follow up

The study by Jacquemart et al. [14] on patients with AVSD, which also includes ostium primum ASD, identified several independent risk factors for atrial arrhythmias. These factors are also relevant for more common ASDs, as they reflect the impact of cardiac remodeling and structural damage. Table 4 summarizes these factors.

The management of ASD in adulthood has evolved towards a proactive approach, with defect closure at the time of diagnosis, before overt symptoms emerge. This has led to a normalization of prognosis for young adults and an improvement in quality of life, functional class, and survival for all adult patients, regardless of age.

However, ASD is associated with a hidden burden of arrhythmias [23]. Patients with small, unrepaired ASDs, even after spontaneous defect closure, show a high prevalence of atrial and ventricular tachyarrhythmias [47]. This suggests that myocardial structural changes induced by early-life volume overload may be partially irreversible and create the substrate for subsequent arrhythmias.

Current guidelines [23] suggest routine follow-up for patients with small ASDs (without evidence of right ventricular enlargement or pulmonary arterial hypertension) every 2–3 years. This follow-up should include a repeated echocardiogram and symptom assessment, especially for arrhythmias, as the presence of atrial arrhythmia can double the risk of congestive heart failure in adults with CHD. Considering the high prevalence of arrhythmias and the natural history of small ASDs, where a potential progression of hemodynamic significance can occur, continuous follow-up for these patients is strongly supported.

It is crucial that follow-up recommendations not only provide information on the late onset of tachyarrhythmias but also on the increased occurrence of late bradyarrhythmias. Since patients with small ASDs and SVT or NSVT were significantly older and diagnosed later than patients with ASD without arrhythmias, traditional risk factors for arrhythmias should also be addressed. Furthermore, in patients with familial ASD, screening for genetic mutations such as NKX2-5, TBX5, and PRRX1 is recommended, which carries an increased risk of early sudden death. It needs to avoid overstating screening in unselected secundum ASD [6].

The risk of stroke is increased in patients with ASD and is not completely eliminated after defect closure. In patients with an open defect, paradoxical embolism is the most commonly suggested mechanism, while in closed defects, the risk of stroke is linked to AF and/or pulmonary venous remodeling after ASD closure [46].

Age at closure is an important predictor of postoperative arrhythmias. Patients with ASD require lifelong specialized care for CHDs in adulthood, particularly those with late ASD closure and incomplete right ventricular remodeling, and patients with ostium primum ASD prone to left atrioventricular valve problems [14].

## 8. Future Perspectives in ASD Management

Future perspectives in the management of the ASD and its arrhythmic complications are centered on a more personalized approach and more sophisticated long-term surveillance. It is becoming crucial to focus on the early identification of patients at high risk of complications, not only through hemodynamic assessment but also via genetic screening, particularly for familial ASD forms with mutations like those in the NKX2-5, TBX5, and PRRX1 genes, which increase the risk of atrioventricular block and sudden cardiac death. A key focus will be defining optimal follow-up strategies for patients with small ASDs, given the finding of a hidden prevalence of arrhythmias in this group. Furthermore, the refinement of advanced imaging techniques, such as CMR, 3D printing, and holograms, will play a growing role in delineating the anatomy of complex defects and in procedural planning. Ultimately, the goal is to optimize the timing of defect closure and transcatheter ablation procedures for arrhythmias to maximize reverse cardiac remodeling, reduce the incidence of post-closure arrhythmias, and ensure all patients with ASD receive continuous, specialized, and lifelong care.

## 9. Conclusions

ASD, though often considered a “simple” defect in anatomical terms, represents a complex condition in adulthood, with significant arrhythmic and conduction complications. The pathophysiology of ASD is closely linked to right heart volume overload, myocardial remodeling, fibrosis, and electrophysiological alterations, which predispose to a wide range of rhythm disturbances, from tachyarrhythmias (AF, AFL, IART, FAT, NSVT) to bradyarrhythmias (AVB, sinus node dysfunction).

Early diagnosis is crucial and relies on standard ECG with specific signs such as IRBBB, crochetage sign, and DTW, supplemented by Holter monitoring and advanced imaging techniques (TTE, TEE, 3DE, CMR, CT). These tools allow for quantification of the shunt and assessment of cardiac remodeling, which are predictive factors for arrhythmic risk.

Treatment must be multidisciplinary and personalized. Defect closure, both transcatheter and surgical, is recommended in the presence of right ventricular overload and before complications manifest. However, the risk of arrhythmias may persist or develop even after closure, especially with advanced age at the time of intervention. Management strategies include pre-closure ablation for AF, post-closure ablation for reentrant tachyarrhythmias, and pacemaker implantation for conduction blocks or sinus node dysfunction.

Ultimately, patients with ASD require continuous and specialized lifelong follow-up, focused on the prevention and management of arrhythmias and other long-term complications. Identifying individual risk factors, including genetic ones, is fundamental for optimizing surveillance and therapeutic strategies.

## Figures and Tables

**Table 1:** Types of Atrial Septal Defects and Their Anatomical and Clinical Features [2].

Type of ASD	Prevalence	Anatomical Localization	Hemodynamic and Clinical Characteristics
**Secundum ASD**	80%	Within the fossa ovalis, there are one or more defects in the septum primum. Generally distant from specialized conduction structures.	The most common type. Size ranges from very small to very large. Usually a left-to-right shunt. Often asymptomatic in children. Can cause dilation of the right atrium and ventricle.
**Primum ASD (partial AVSD)**	15%	Between the inferior margin of the fossa ovalis superiorly and the AV valves inferiorly. No ventricular component. Characterized by a common AV junction with two distinct valvular orifices and almost always anomalous AV valves.	Associated with AV valve abnormalities, often a mitral fissure. The defects are usually significant. Causes left axis deviation on the ECG. Higher risk of AV block.
**Sinus Venosus ASD**	5–10%	Typically located in the mouth of one of the vena cavae. The superior defect is more common (5%), due to a lack of tissue separating the right superior pulmonary vein from the superior vena cava. The inferior defect is rare (<1%).	It is often associated with anomalous pulmonary venous drainage. The proximity to the crista terminalis and the sinus node increases the risk of sinus node dysfunction. It almost always requires surgical repair.
**Coronary Sinus Defect**	<1%	Result of partial or complete unroofing of the tissue separating the coronary sinus from the left atrium, allowing a shunt through the defect and the coronary sinus orifice.	Rare. Commonly associated with persistent left superior vena cava (Ragbhib syndrome). Rhythm considerations beyond the effect of the left-to-right shunt have not been widely reported. Requires surgical repair.

**Table 2:** The features of patients with pacemaker implantation.

Cohort/Registry	Age at Closure (or Mean Age)	Follow-up Duration/ Temporality	Endpoint and Incidence (%)
Muroke et al. [33]	>25 years	Overall prevalence (not specified if early or late)	AVB: 6.4% in closed defects. >60 times higher risk for AVB compared to healthy controls.
Bergmann et al. [34]	Advanced age at closure (a factor that may influence the device group)	Prevalence (not specified, but highlights the gradient)	AV Block Prevalence: Catheter Closure: 13%; Surgical Closure: 5%
Albæk et al. [15]	Mean age at PM implantation: 32 years	Late events	PM required: 3.4%
Chen et al. [9]	N/A	N/A	LAFB: 5.8% in closed defects.

ASD: atrial septal defect; AVB: Atrioventricular Block; LAFB: Left anterior fascicular block; PM: Pacemaker.

**Table 3:** ECG clues to ASD by type.

Electrocardiogram Clues	Sensibility	Specificity	Likelihood Ratio Positive
Crochetage in at least 1 inferior lead (II, III, aVF)	73% (0.73)	92% (0.92)	9.125
Crochetage in all 3 inferior leads (II, III, aVF)	Not directly reported	≥95%	Extremely high
IRBBB + Defective T Wave	≈87%	100%	Virtually infinite

**Table 4:** Risk Factors for Atrial Arrhythmias in Patients with AVSD (Adapted from Jacquemart et al. [27]).

Risk Factor	Odds Ratio (OR)	Confidence Interval (95% CI)	*p*-Value
Age (for every 5-year increment)	1.4	1.2–1.6	<0.001
Number of cardiac surgeries	4.1	2.5–6.9	<0.001
Left atrial dilation	3.1	1.4–6.8	0.005
Right atrial dilation	4.1	1.7–10.3	0.002
Moderate or severe left AV valve regurgitation	3.7	1.2–11.7	0.021
